# Papillary Endothelial Hyperplasia (Masson Tumor) of the Hand. Surgical and Pathological Consideration from Seven Cases Using New Vascular Markers

**DOI:** 10.1007/s12253-020-00838-8

**Published:** 2020-07-15

**Authors:** Bernadett Bettina Patai, Nora Peterfy, Noemi Szakacs, Zoltan Sapi, Judit Reka Hetthessy

**Affiliations:** 1 Department of Traumatology, Military Health Centre, Budapest, Hungary; 2 Department of Traumatology and Hand surgery, Saint John’s Hospital, Budapest, Hungary; 3 Department of Orthopedics, Semmelweis University, Budapest, Hungary; 4 First Department of Pathology and Experimental Cancer Research, Semmelweis University, Budapest, Hungary

**Keywords:** Hand, Soft tissue lesion, Vascular tumor, Vascular malformation, Intravascular papillary endothelial hyperplasia, IPEH, Masson tumor

## Abstract

Although papillary endothelial hyperplasia may occur at almost any site, one of the most common sites is the hand. It is generally regarded as a reactive vascular proliferation i.e. exuberant form of organizing thrombus. Diagnosis of Masson tumor can be challenging due to its close clinical, radiological and even histopathological resemblance to angiosarcoma. We present seven cases of Masson tumor of the hand; wanting to reveal its nature using new vascular markers and discuss the treatment options and expected outcomes, present clinical and radiological features that may aid diagnosis and also offer treatment plans. A multicenter retrospective study was performed between January 2014 and November 2019. Immunohistochemical stains of Glut1, WT1, ERG, CD31 and alpha smooth muscle actin (ASMA) were performed on each cases. We found seven cases during the examined period. 4 out of 7 cases were women. All lesions occurred in the hands. 3 out of 7 cases appeared in a previously present vascular malformation. All cases were treated with surgical excision and the diagnosis of papillary endothelial hyperplasia was made by histology. Pre-operative testing (radiograph/MRI/US/fine needle aspiration biopsy) did not suggest the diagnosis of Masson tumor; however, aspiration cytology could rule out malignancy. The proliferative endothelial cells proved to be Glut1 negative and WT1 positive and the accompanying pericytic cells were ASMA positive in all cases. Though Masson tumor is a rare vascular lesion in the hand among other vascular tumors, it should be considered in the differential diagnostics even in the case of previously existing vascular malformation. WT1 positivity of the endothelial cells and the accompanying pericytic cells raises the question whether the initially reactive endothelial proliferation may transform into a true benign vascular tumor.

## Introduction

Masson tumor is a rare malformation of skin and subcutaneous tissues and was first described by Pierre Masson in 1923 [19]. Intravascular Papillary Endothelial Hyperplasia (IPEH), also called Masson’s Tumor/ Masson Tumor, is an entity that is described as appearing as either a reactive or a neoplastic process and as a benign entity [30]. It presents with exuberant organization and recanalization of thrombi [17, 25]. It can appear in normal vessels but also in varices, hemorrhoids, previously-existing vascular tumors and in hematomas [31]. They are rare vascular lesions among other vascular tumors in the hand and may pose a differential diagnostic problem making it difficult to plan surgery and aftercare for the patient. It is a slow growing lesion that is usually characterized by palpable and visible swelling [10, 12, 18]. Symptoms may vary, palpable mass, pain, swelling, and limited range of motion may occur. Usually these lesions are treated by orthopaedic, trauma or hand surgeons but it is not rare for patients to be seen by general or plastic surgeons. These lesions account for approximately 2%–4% of vascular tumors of the skin and subcutaneous tissues [27, 30, 15]. An unusual case with rapid growth was recently reported by Corni et al., [8] but they did not observe recurrence or complications at a 6 months follow-up. Furthermore, though 15% of recurrence rate was noted in recent literature; [8, 28] all of these cases had a benign natural history.

The differential diagnosis of vascular tumors in the musculoskeletal system may be challenging because radiologists are usually able to identify only the vascular nature of the lesion, but it is difficult to differentiate IPEH from other malignant vascular neoplasms [32, 33]. Masson tumors may present with features of benign or malign tumors e.g.: hemangiomas, hemangioendotheliomas and other rare vascular neoplasms, including angiosarcomas [3, 33, 34].

In this work we present seven cases of Masson tumor of the hand; wanting to reveal its nature using new vascular markers and discuss the treatment options and expected outcomes and present clinical and radiological features that may aid diagnosis and also offer treatment plans.

## Methods

A multicenter retrospective study was performed at the Department of Orthopedics Semmelweis University, at the Department of Orthopedics-Traumatology of Saint John’s Hospital, at Budaörs Healthcare Center and 2nd District Municipal Health Service between January 2014 and November 2019. Patients who had undergone surgery at these institutions for soft tissue growths of the hand and wrist during this period were included. We filtered patients according to the results of the histopathological examination. Data from the patients whose histopathological diagnosis was Masson tumor will be presented.

*Immunohistochemical* staining was performed with Bond Max™ Autostainer (Leica Biosystems Newcastle, Newcastle, UK). Tissue sections of 3 μm thickness were cut from the blocks, followed by deparaffinization in xylene and retrieval using either the Bond Epitope Retrieval Solution 1 (pH ∼ 6) or the Bond Epitope Retrieval Solution 2 (pH ∼ 9) (Leica Microsystems, Wetzlar, Germany) at 99–100 °C for 20–30 min, and immunostained using a monoclonal mouse anti-ASMA antibody (clone:1A4; 1:400; Agilent DAKO, USA California Santa Clara), monoclonal mouse anti-human ERG (clone EPR 3864; ready to use; Ventana, Roche Diagnostics USA Indianapolis), monoclonal mouse anti-human WT1 (clone 6F- H2; 1:300; BioSB, USA Santa Barbara CA), monoclonal mouse anti-human CD31, (clone JC70A; 1:300; Agilent Dako, USA California Santa Clara) and polyclonal mouse anti-human Glut1 (1:100; Cell Marcque, USA Rocklin California). Sections were incubated with the primary antibody for 25 min, followed by using the peroxidase/DAB Bond Polymer Refine Detection System (Leica Microsystems) for visualization.

## Results

In the above-mentioned period, histopathological diagnosis was Masson tumor in all 7 cases. Table 1 summarizes patient age, gender, exact location of the lesion and duration of symptoms. The average patient age was 40 years (24–71). 4 patients were female and 3 were male. The lesions appeared on the left side in 4 cases and in 3 cases on the right side. 2 patients had vascular lesions since childhood, they had complaints for 6 or 8 months before their surgery. In 4 patients, the tumor occurred 4, 6, 24 and 24 months earlier and their complaints started at 4, 2, 24 and 24 months before their surgery. One patient noticed the lesion 2 days earlier and had had complaints for 1 week before surgery. Previous treatments and the results of pre-operative radiological examinations of the patients are detailed. Sclerotization was performed for Patients No.1 and 3 and surgical excision for Patients No. 3 and 4. We have to emphasize, regarding the pre-operative examinations, that the earlier histopathological diagnosis of Patient No.1 was a hemangioma; the ultrasound examination of Patient No. 2. presumed a ganglion and the FNAB suspected hemangioma; the MRI examination of Patient No.3. presumed hemangioma; 1 patient had previous histological examination and MRI; 1 patient had US and FNAB examination, (radiograph); 1 patient had MRI, (radiograph) and 4 patients (Nos. 4, 5, 6 and 7.) underwent clinical examination only before the operation, their diagnosis was established through the histological examination. The Masson tumor appeared on the basis of a hemangioma for Patients No. 1 and 3; Patient No. 4 presumably had a different type of pre-existing vascular diagnosis, however, no exact histology was available. The lesions were also analyzed according to whether they formed on a pre-existing vascular lesion, or if they could be considered de novo. Patient No. 2 and 5 had de novo type Masson tumors. Planned surgical care was also logged in this table. Marginal excision was performed in all cases. No complications were observed beside a transient ischemia around the 1st digital nerve in patient No 3. The follow-up ranged between 3 and 66 months, with an average of 15.42 months. In this period, recurrences were not observed. All patients had good function and were able to return to previous activities and resume their work. Histopathological diagnosis was Masson tumor in all 7 cases and concerning the pathological marker results GLUT1 negative; WT1, CD31, ERG, ASMA positive (summary is in the table).

**Table 1:** Summary of patient age, gender, location of lesion, appearance of the lesion, duration of symptoms**,** previous treatments, radiological examinations, surgical care, postoperative complications, recurrences, pathological markers

Clinicopathology							
Patient number	1	2	3	4	5	6	7
AGE (YEARS OLD)	26	30	24	36	71	31	61
GENDER	female	female	male	male	female	male	female
SIDE, location	2nd metacarpo-phalangeal jointl, palmar side of the right hand	Proximal phalanx of the left thumb	1st metacarpo-phalangeal joint, palmar side of the left hand	Distal phalanx of the right thumb	Distal phalanx of the left ring finger, dorsoradial side	Distal phalanx of the left ring finger, dorsal side	Middle phalanx of the right ring finger, palmar side
HOW LONG HAS THE PATIENT HAD A VASCULAR LESION	since childhood	for 4 months	since childhood	for 6 months	for 24 months	for 24 months	for 2 days
LENGTH OF COMPLAINTS	6 months	4 months	8 months	2 months	24 moths	24 moths	for 1 week
Previous treatmenTs	sclerotization 1x	–	sclerotization 1x, surgical treatment 5x	surgery 1x	–	–	–
Symptoms	palpable mass, grew in size; tenderness to palpation	palpable mass, grew in size; tenderness to palpation, limited range of motion	palpable mass, grew in size; tenderness to palpation, limited range of motion	discomfort, palpable mass, tenderness	discomfort, palpable mass	palpable mass, changing in size on occasion; no local tenderness	discomfort, palpable mass, tenderness
Pre-operative diagnosis	haemangioma	ganglion haemangioma	haemangioma	not obtained	not obtained	not obtained	not obtained
Diagnosis bases on	prev. Histopathological diagnosis	US FNAB	MRI	clinical examination	clinical examination	clinical examination	clinical examination
De novo / secondary	previous haemangioma	de novo	previous haemangioma	previously histological diagnosis was not performed	de novo	de novo	de novo
Surgical care	marginal excision	marginal excision	marginal excision	marginal excision	marginal excision	marginal excision	marginal excision
Complications	–	–	transient paraesthesia around the 1st digital nerve, which resolved	–	–	–	–
Lenegth of follow up (Months)	12	12	3	6	3	6	66
Recurrence	–	–	–	–	–	–	–
functional results	hand function improved (circulation, sensation, motion = CSM, in order)	hand function improved,CSM in order	hand function improved, transient	hand function improved, (CSM in order)	hand function improved, (CSM in order)	hand function improved, (CSM in order)	hand functions improved, (CSM in order)
Pathological markers (Glut1, WT1, CD31, ERG, ASMA)	GLUT1 negative; WT1, CD31, ERG, ASMA positive	GLUT1 negative; WT1, CD31, ERG, ASMA positive	GLUT1 negative; WT1, CD31, ERG, ASMA positive	GLUT1 negative; WT1, CD31, ERG, ASMA positive	GLUT1 negative; WT1, CD31, ERG, ASMA positive	GLUT1 negative; WT1, CD31, ERG, ASMA positive	GLUT1 negative; WT1, CD31, ERG, ASMA positive

### Short Clinical Summary of Patients Who Has Entirely Documented Pictures

**Fig. 1:**
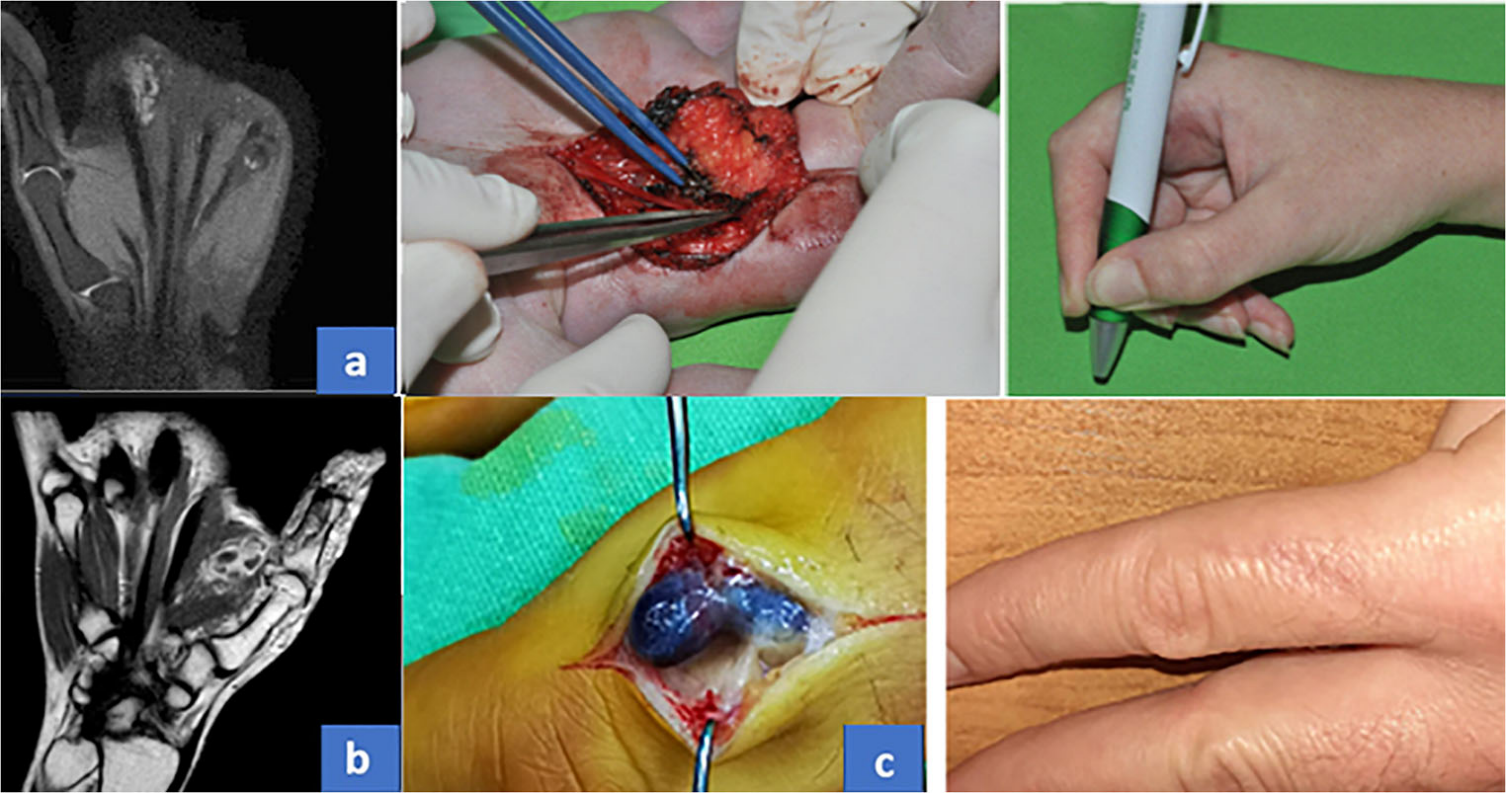
**a** From left to right: T2-weighted anterio-posterior MRI image of Patient No 1., Surgical excision, Post-operative function. **b** T2-weight AP MRI of Patient No 3. **c** Surgical excision performed for Patient No 6., result at 6-months follow-up

26-year-old female patient had a pre-existing vascular malformation on the right hand since her childhood. Surgical excision was performed once in 2011 and proved to be hemangioma based on the histopathological report. The preoperative MRI **(**Fig. 1a**)** displayed the recurrence of the hemangioma. Surgical excision was performed **(**Fig. 1a**)**. She had a good recovery **(**Fig. 1a**)**, there were no complications from surgery, circulatory or sensation disturbances did not appear.

24-year-old male patient had a pre-existing hemangioma on the left hand since childhood. Pre-operative radiological workup was performed (radiograph, Ultrasound, MRI). MRI **(**Fig. 1b**);** indicating a hemangioma. Surgical excision was performed. Histological examinations diagnosed Masson tumor.

31-year-old male patient noticed a mass growing on his left ring finger. Based on patient complaints and physical examination this was presumed to be a hemangioma. Surgical excision was performed **(**Fig. 1c**)**. The histological examination diagnosed the lesion as a Masson tumor. No recurrence appeared during the 6-month follow-up period **(**Fig. 1c**)**.

### Pathological Findings

**Fig. 2:**
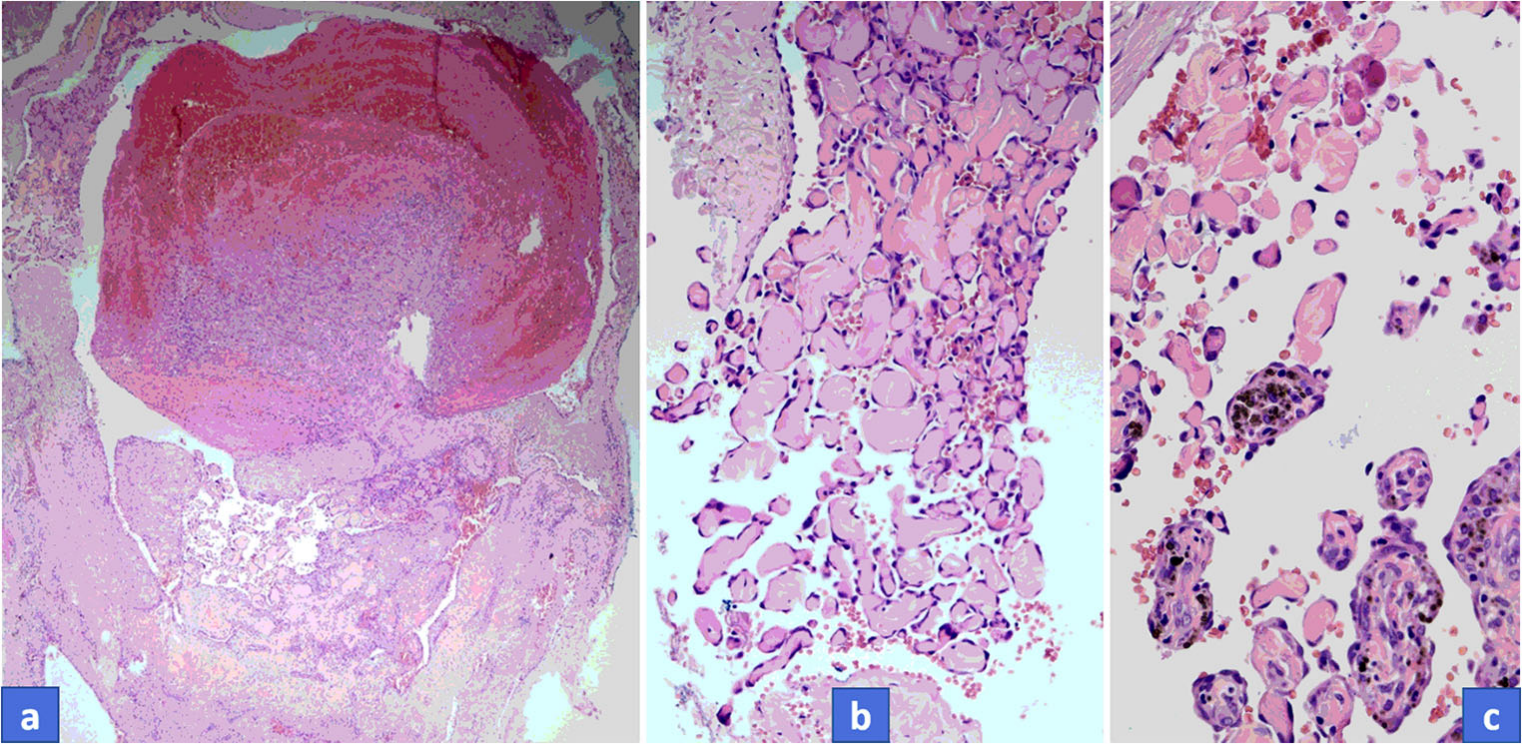
**a**, Characteristic overview of Masson tumor: dilated vessel with clotted blood and papillary endothelial proliferation. **b**, Papillae are composed of a single layer of endothelium with collagenized core. **c**, Hemosiderin deposits in connective tissue could be observed in almost all cases

**Fig. 3:**
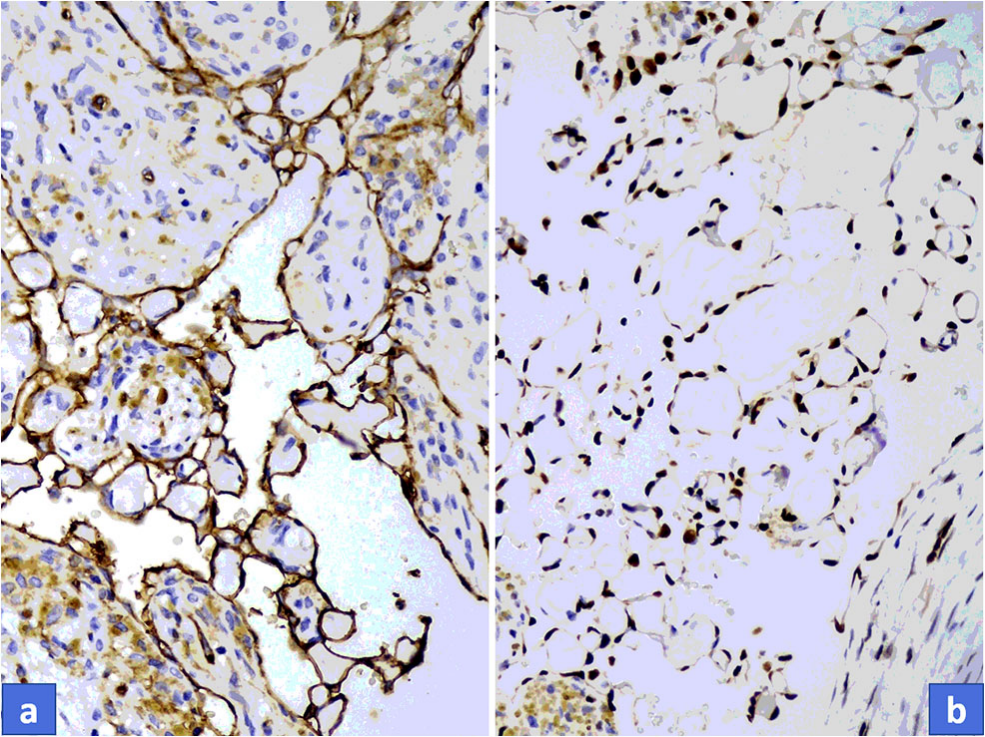
Strong cytoplasmic CD31 **(a)** and nuclear ERG **(b)** positivity could be detected on endothelial cells, but it is also evident that other cellular components are present in the tumor. (Immunohistochemical stain with CD31 and ERG)

**Fig. 4:**
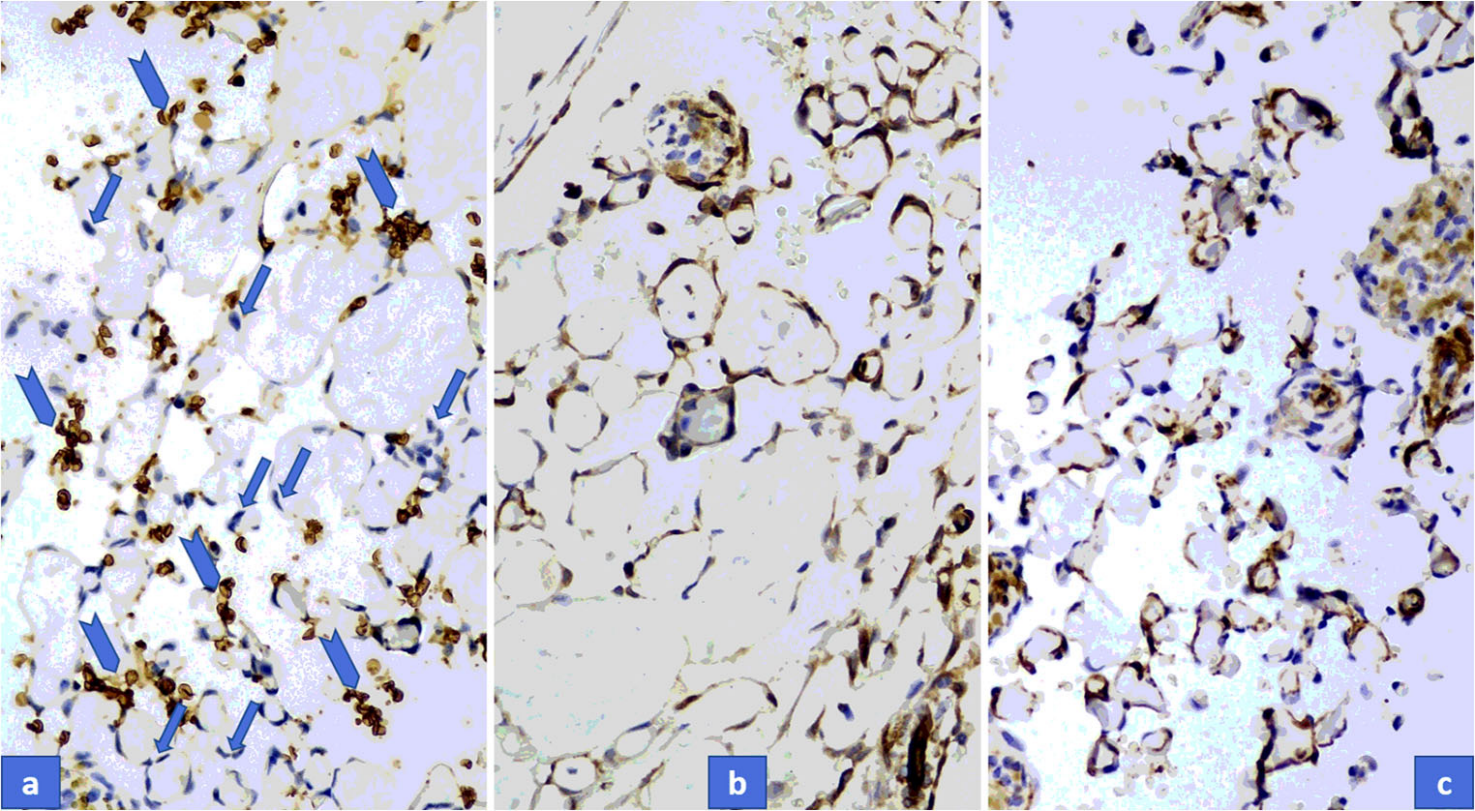
Glut1 is negative in endothelial cells (arrows), only red blood cells are positive (arrowheads) (**a**). WT1 is clearly positive on endothelial cells (**b**), while ASMA displays pericytes (**c**). (Immunohistochemical stain with Glut1, WT1, and ASMA)

All seven tumors displayed similar histological pictures. Lesions appeared in a dilated vessel or in pre-existing hemangioma, such as in cases of No. 1 and No. 3 (Fig. 2a). A papillary proliferation of endothelial cells was typical in all cases along with more or less clotted blood. Papillae are composed of a single layer of endothelium with collagenized core; sometimes forming an anastomosing network of vessels (Figs. 2b-c). More or less connective tissue was also obvious and a separated layer of pericytic cells along with endothelial cells could also be observed, though it was much more appreciated with ASMA immunostaining. Hemosiderin deposits in connective tissue or in cellular elements were also a common finding. Cytoplasmic CD31 and nuclear ERG positivity (as vascular markers) displayed the endothelial cells (Fig. 3) and ASMA positivity proved the presence of pericytic population with close tightness of endothelial cell (Fig. 4c). However, the pericytic layer was not always continuous, sometimes only focal, but in all cases the accompanying pericytes could be observed. While the Glut1 was consistently negative in endothelial cells the WT1 proved to be positive in the vast majority of endothelial cells (Figs. 4 a-b).

## Discussion

IPEH was originally designated as “vegetant intravascular hemangioendothelioma” by Masson and he regarded it as a true neoplasm with intermediate malignancy (applying the recent WHO nomenclature). Although it turned out very soon after the initial description that this is a benign lesion, nevertheless the debate whether it is a true neoplasm or a reactive vascular proliferation continued. However, even if many pathologists accepted the “reactive” nature of this peculiar lesion, clinically it appears as a true neoplasm, especially because of its relatively high recurrence rate.

Pathogenesis of the tumor is not clear. It may appear de novo or they may form in association with or on the basis of other pre-existing vascular lesions or it can be in extravascular form which arises in a hematoma. Other investigators [21] have argued that thrombosis occurs prior to papillary growths and the following fibrin deposition acts as a substrate for the IPEH development. Fibroblast Growth Factor and fibrinous deposits have also been proven to induce the process [25, 30]. Levere et al., [16] however, proposed an autocrine etiology of post-traumatic IPEH, involving the fibroblast growth factor (FGF) secretion. The macrophages that reach the site of trauma release the FGF, which triggers IPEH; the endothelial proliferating cells, on their turn, release more FGF, thus activating a positive feedback loop of endothelial proliferation. [3]

Recently, using new vascular markers combined with vascular flow parameters [23], a new classification proposal is emerging. However, no systematic examination of Masson tumor with these new vascular markers has happened so far. Currently, it is agreed that true capillary hemangioma, which is capable for spontaneous regression, should be Glut1 positive regardless of its morphological appearance [24]. Therefore, we wanted to examine the Glut1 status of our cases and no Glut1 positivity was found at all. Spontaneous regression can be characteristic for some benign tumor and also for some reactive proliferation but in our cases, we found negativity which is against indirectly to the proliferative nature of the Masson tumor. Cytoplasmic WT1 positivity is characteristic for normal endothelial cells and can be observed in many benign vascular tumors. Interestingly, Timar et al. found that malignant vascular tumors are more frequently positive with WT1 as compared to benign ones [2]. On the other hand, Al Dhaybi et al., evaluating the expression of WT1 in 126 vascular lesions, found that WT1 positivity allows the distinction of vascular tumors from vascular malformations [29]. WT1 positivity of our cases proves that Masson tumor is not a vascular malformation and also favours the concept of true neoplasm. Pericytes have an important role in the formation of vascular tumors. Usually benign vascular tumors can be characterized by the accompanying pericytes, however, the lack of a pericytic layer is quite specific for the malignant vascular tumors. Because Masson tumor is a benign entity and pericytes may appear both in proliferative and benign vascular lesions (but it is more characteristic for benign ones), the presence of pericytic layer also speaks for the neoplastic nature.

Considering the long-standing condition of Masson tumor, the relatively high recurrence rate, the appearance in preexisting vascular tumors, the Glut1 negativity and WT1 positivity and the accompanying pericytic layer and furthermore the constant positivity with different vascular markers (CD31, ERG), it seems that Masson tumor begins as a proliferative process but with time it transforms into a true benign neoplasm. It is also important to consider Masson tumor as a benign tumor because the possibility for further transformation into a malignant one cannot be excluded, however, there has been no report of this phenomenon so far.

In our series, pre-operative radiological examinations were unable to establish the exact diagnosis of Masson’s tumor - even the FNAB did not point directly to this lesion. Our 7 cases also demonstrate that there are 2 major etiological factors behind the IPEH. The lesion can develop de novo (5 out of 7 cases) or on the basis of pre-existing vascular malformations (hemangiomas, 2 out of 7 cases). The clinical appearance can be from no symptom to a limited of range of motion. In hand surgery, it is very important to plan the appropriate surgical solution to prevent recurrence.

The main problem during the differential diagnostic process is not that Masson’s tumor may be misinterpreted as a different benign malformation e.g.: hemangioma, but that due to radiological appearance and sometimes features of FNAB, it may be over-diagnosed. This may open the gate for overtreatment if it is presumed to be a malignant tumor (e.g.: angiosarcoma) [20, 22].

Masson’s tumor usually occurs in adults aged 30–40 years and is slightly most common in women, just as hemangiomas, suggesting a hormonal factor in development of this kind of lesions [6, 9]. Females are slightly more affected than males (ratio 1.14: 1) [9, 13] according to the literature. In our series, 4 out of 7 patients were female and the average age was 40 years. According to the literature, Masson’s tumor is a rare lesion in the hand (among other vascular tumors) [1], but we found 7 cases (5 of out 7 cases were on the fingers (thumb and ring finger); 2 of out 7 cases were on the thenar during the examined period, which is relatively high, considering that Masson’s tumor accounts for approximately up to 2%–4% of benign and malign vascular tumors of the skin and subcutaneous tissues [27, 30]. In the literature, the frequency of Masson tumor on the hand is about 7%. [5, 30]

In 2 of out 7 cases, the Masson tumor appeared in a previously present hemangioma. This is relatively high, but there is no exact ratio about it in the literature.

All 7 cases included a palpable mass and tenderness to palpation. In 4 cases, the lesion demonstrated growth which resulted in impaired finger function. Remarkably, symptoms can enhance drastically on the hand, compared to other regions of the body, since even a small lesion may interfere with harmonious hand function due to discomfort and a limited range of motion.

Marginal surgical excision is the hallmark of treatment for these lesions in the hand. Total excision would theoretically reduce the risk of recurrences. However, this may be technically difficult to perform due to location and the tumor being interwoven with the vasculature of the finger. We encountered this limitation when performing excision of expansive lesions. [7, 13] As an alternative treatment, sclerotherapy may be considered. However, due to the characteristics of the vascular anatomy of the fingers, this procedure may carry the risk of circulatory problems and even necrosis [14] of the fingers in certain cases. In our series, 2 patients had sclerotherapy but as the lesion progressed on the fingers, this was no longer an acceptable option.

FNAB was performed in one of our cases and it did not raise the possibility of Masson tumor, but suspected hemangioma. In 2 other patients who underwent diagnostic tests (US, MR) before surgery, hemangioma was suspected too. Unfortunately, neither the clinical examination, nor the FNAB could give an absolutely accurate diagnosis. The final exact diagnosis was given by postoperative histology in all 7 cases. It is difficult to plan the surgery in the absence of an accurate diagnosis, but if there is no evidence for malignancy, as it happened in our cases, the proper route can be chosen. During surgery, care should be taken of the surrounding anatomical structures; especially with digital terminal arteries because they are responsible for the blood supply of the fingers and as these may be interwoven with the vessels of the vascular tumor. Dissecting them from the vessels of the Masson tumor may be quite an intricate task.

In each case, a discussion with the patient and an individual consultation must precede the operation as these are slowly increasing lesions which cause progressive symptoms over time. Most of the symptoms are increasing pain, discomfort and consequently, a limited range of motion. Once the diagnosis has been made, patients also need to be informed regarding expected outcomes and recurrence rates, the necessity of radiological follow-up or the possibility of further operations. We plan to follow our cases every 3–6 months for 1 year, and once a year for the next 3–4 years. If clinical progression is suspected, the patient is referred for further imaging studies. Patients are also advised to register for a check-up if they notice any growth or in case if new symptoms appear. According to the literature, the expected recurrence rate of these lesions is around 15%. It has been described that if IPEH arises in a pre-existing vascular lesion, the recurrence rate depends on the technical difficulties as to how someone can remove the original vascular tumor. In our series no recurrence appeared during follow-up. However, this period was not a long-term one. We expect especially that in the patients who had pre-existing vascular lesions, recurrence may appear in the subsequent years, therefore, radiological follow-up is recommended [4, 11, 12, 18, 26]. Physiotherapy to regain range of motion and strength may be necessary.

## Conclusion

Though Masson tumor is a rare vascular lesion in the hand among other vascular tumors, it should be considered in the differential diagnostics even in case of a previously existing vascular malformation. It is important to have the most conclusive preoperative diagnosis. Therefore, preoperative examinations such as radiography, MRI/ultrasound and also fine needle biopsy can be necessary to aid the diagnosis and differential diagnosis of Masson tumor or at least exclude malignancy. Depending on size, location, symptoms and rate of growth [17, 21], surgical excision remains the hallmark of treatment options. As it is a benign lesion, marginal excision is adequate, however, incomplete excision may influence recurrence rates. Due to its dimensions and localization, the entire lesion may not be possible to remove in certain cases and therefore progression and recurrence may be expected. We also concluded that Masson tumor begins as a proliferative process, but with time it transforms into a true benign neoplasm based on the following clinical and pathological findings: the long-standing condition of Masson tumor, the relatively high recurrence rate, the appearance in preexisting vascular tumors, the Glut1 negativity, WT1 positivity, the accompanying pericytic layer, and the constant positivity with different vascular markers (CD31, ERG).

## Abbreviations

CSM: circulation
FNAB: fine needle aspiration biopsy
IPEH: Intravascular Papillary Endothelial Hyperplasia
ROM: Range of motion
US: ultrasound
